# In vitro and in vivo evaluation of an oral microemulsion formulation of *Centaurea lycopifolia* Boiss. Et Kotschy extract for analgesic and anti-inflammatory effects in a carrageenan-induced model

**DOI:** 10.1007/s10787-026-02124-6

**Published:** 2026-02-09

**Authors:** Sonia Ebrahimi, Umay Merve Güven Bölgen, Serpil Demirci Kayıran, Tilbe Çevikelli, Mehmet Boğa, Fazilet Aksu

**Affiliations:** 1 Department of Medical Pharmacology, Faculty of Medicine, Near East University, Nicosia, TRNC Turkey; 2 Department of Pharmaceutical Technology, Faculty of Pharmacy, Çukurova University, Adana, Turkey; 3 Department of Pharmaceutical Botany, Faculty of Pharmacy, Çukurova University, Adana, Turkey; 4 Department of Pharmaceutical Technology, Faculty of Pharmacy, Bahçeşehir University, Istanbul, Turkey; 5 Department of Analytical Chemistry, Faculty of Pharmacy, Dicle University, Diyarbakır, Turkey

**Keywords:** Centaurea lycopifolia, Analgesic effect, Anti-inflammatory effect, Microemulsion formulation, Carrageenan-induced inflammation

## Abstract

**Purpose:**

*Centaurea lycopifolia* Boiss. et Kotschy (Asteraceae) is traditionally used in folk medicine for wound healing. This study aimed to develop and pharmacologically evaluate a novel oral microemulsion containing *C. lycopifolia* extract, focusing on its analgesic and anti-inflammatory effects in rodent models.

**Methods:**

The extract, obtained from aerial parts of the plant, was characterized by LC-MS/MS. A microemulsion formulation was developed for oral administration. Antinociceptive activity was evaluated via hot plate (HP) and tail flick (TF) tests to assess central and spinal effects, respectively. Anti-inflammatory activity was assessed using carrageenan-induced paw edema, quantified by plethysmometry and Randall–Selitto tests.

**Results:**

LC-MS/MS analysis identified quinic acid, chlorogenic acid, and protocatechuic acid as major phytoconstituents. In both HP and TF tests, the *C. lycopifolia* microemulsion demonstrated significantly stronger antinociceptive effects than aspirin. Similarly, its anti-inflammatory activity was comparable to aspirin. These pharmacological effects are possibly associated with the synergistic actions of the phenolic acids present in the extract.

**Conclusions:**

The *C. lycopifolia*-loaded microemulsion exhibited strong in vivo analgesic and anti-inflammatory activity, supporting its potential as a phytopharmaceutical candidate for inflammatory pain. The use of both central and peripheral pain models provided a robust pharmacodynamic basis for its therapeutic potential. Overall, these findings highlight the relevance of phenolic-rich phytochemicals in oral delivery systems for inflammation-related disorders.

## Introduction

Rheumatic diseases are conditions due to inflammation in bones, muscles, and joints. More than one hundred rheumatic diseases have been recognized, which vary from rare to common. These diseases are defined as multi-system diseases because they affect other systems as well as muscles and joints. The most prominent features are pain and limitation of movement around one or more joints. While the cause of rheumatic diseases is unknown, genetic, immune, and environmental factors are thought to play a role. Common rheumatic diseases include rheumatoid arthritis and osteoarthritis. Arthritis is an inflammatory disease characterized by pain, swelling, redness, and loss of joint function (Radu and Bungau 2021).

The International Association for the Study of Pain (IASP) defines human pain as “an unpleasant sensory and emotional experience associated with or defined in terms of actual or potential tissue damage” (Cohen et al. 2018). Analgesia is the inability to feel pain. The leading pain relief method is the use of analgesics, which are one of the most commonly used drugs, along with antibiotics. Analgesics include opioid drugs such as morphine and nonsteroidal anti-inflammatory drugs (NSAIDs) such as aspirin and ibuprofen (Alorfi 2023). Analgesics are frequently used but often misused. When used as directed, they control pain in 85% of patients. Symptomatic pain management aims to improve quality of life. The pharmacological properties of the drugs, pain intensity, and psychological characteristics of the patient are also important in choosing the right analgesic. NSAIDs are the preferred analgesic for mild to moderate pain and inflammation and are the primary treatment for pain. However, long-term use of nonsteroidal drugs can cause harmful side effects such as ulceration, bleeding, and cardiovascular and gastrointestinal irritation. Similarly, opioids, which are used as strong analgesics, can lead to addiction and side effects. New drugs for pain and inflammation treatment including plant-derived natural products such as flavonoids, sterols, polyphenols, alkaloids, tannins, and terpenes have garnered attention recently due to their broad pharmacological properties (Gouveia et al. 2019; Goyal et al. 2023; Hasan et al. 2024).

Turkiye has 109 endemic species out of 180 species in the genus Centaurea (Asteraceae) (Uysal et al. 2016). Traditional folk medicine utilizes this genus for its expectorant, antifungal, antiulcerogenic, antioxidant, antiplasmoidal, antiprotozoal, antipyretic, antibacterial, anticancer, antidiarrheal, antidiabetic, antirheumatic, anti-inflammatory, antipyretic, diuretic, menstrual, hypotensive, choleretic, digestive, and stomachic properties (Gözcü et al. 2024; Paniagua-Zambrana et al. 2024).

Herbal medicines have become popular in developed countries. Their effects are typically not a result of being natural but are instead determined by the pharmacological properties and dosage of their active ingredients. Analysing these components is essential for standardizing herbal medicines (Kumari and Kotecha 2016). It is necessary to develop a suitable drug delivery system to raw materials (plant-based or chemical) into the body through various routes (oral, transdermal, nasal, etc.). It is stated that nanotechnological drug delivery systems are more successful in addressing issues such as solubility problems, low bioavailability, and stability commonly encountered in traditional drug delivery systems (Teja et al. 2022; Wani et al. 2023).

Microemulsion systems are characterized by low interfacial tension, large interfacial area, small droplet size, low viscosity, and a strong solubilization capacity for lipophilic, hydrophilic, and amphiphilic compounds. Due to the presence of both water and oil phases, it can easily dissolve various active compounds in herbal extracts. There are numerous studies reporting both nanoemulsion and microemulsion formulations incorporating herbal extracts (Maroof et al. 2023; Sanguansajapong et al. 2022). Microemulsions and nanoemulsions, although often used interchangeably in the literature, represent fundamentally different colloidal systems with distinct physicochemical properties. Microemulsions are thermodynamically stable, spontaneously forming isotropic dispersions stabilized by high concentrations of surfactants and co-surfactants, typically exhibiting droplet sizes between 10 and 300 nm. In contrast, nanoemulsions are kinetically stable systems that require high-energy homogenization to form, and their stability is governed by kinetic barriers rather than thermodynamic favourability. Nanoemulsions generally feature droplet sizes ranging from 20 to 500 nm and show a propensity for Ostwald ripening or coalescence over time unless optimized. While microemulsions form spontaneously due to ultralow interfacial tension, nanoemulsions do not self-assemble and instead rely on mechanical energy input. This terminological distinction is critical, as the formulation strategies, stability mechanisms, and application fields of the two systems differ significantly despite superficial similarities in size range (Bolgen et al. 2025). A literature survey shows that although many microemulsion and nanoemulsion systems have been developed for the oral delivery of plant extracts, no published paper reported on the formulation of an oral microemulsion containing Centaurea lycopifolia. An oral microemulsion spray, for an instance applied for Centella asiatica extract was successfully developed for improving wound healing in mouth ulcer pointing towards the use of microemulsion systems for herbal actives (Sanguansajapong et al. 2022). Similarly, microemulsions have been employed to enhance oral bioavailability and targeted delivery of compounds including lycopene and curcumin along with other herbal extracts (Hu et al. 2012; Guo et al. 2019). Nevertheless, investigations of Centaurea lycopifolia spp had been mainly conducted on its phytochemical content and biological activities as an antioxidant and enzyme inhibitor with no reports on sophisticated oral delivery systems lie microemulsions (Zengin et al. 2018). To our knowledge it is the first report on the development and characterization of an oral microemulusion formulation of *C. lycopifolia* that contributes to fill a gap in literature, providing new perspectives to improve its therapeutic action.

**Fig. 1:**
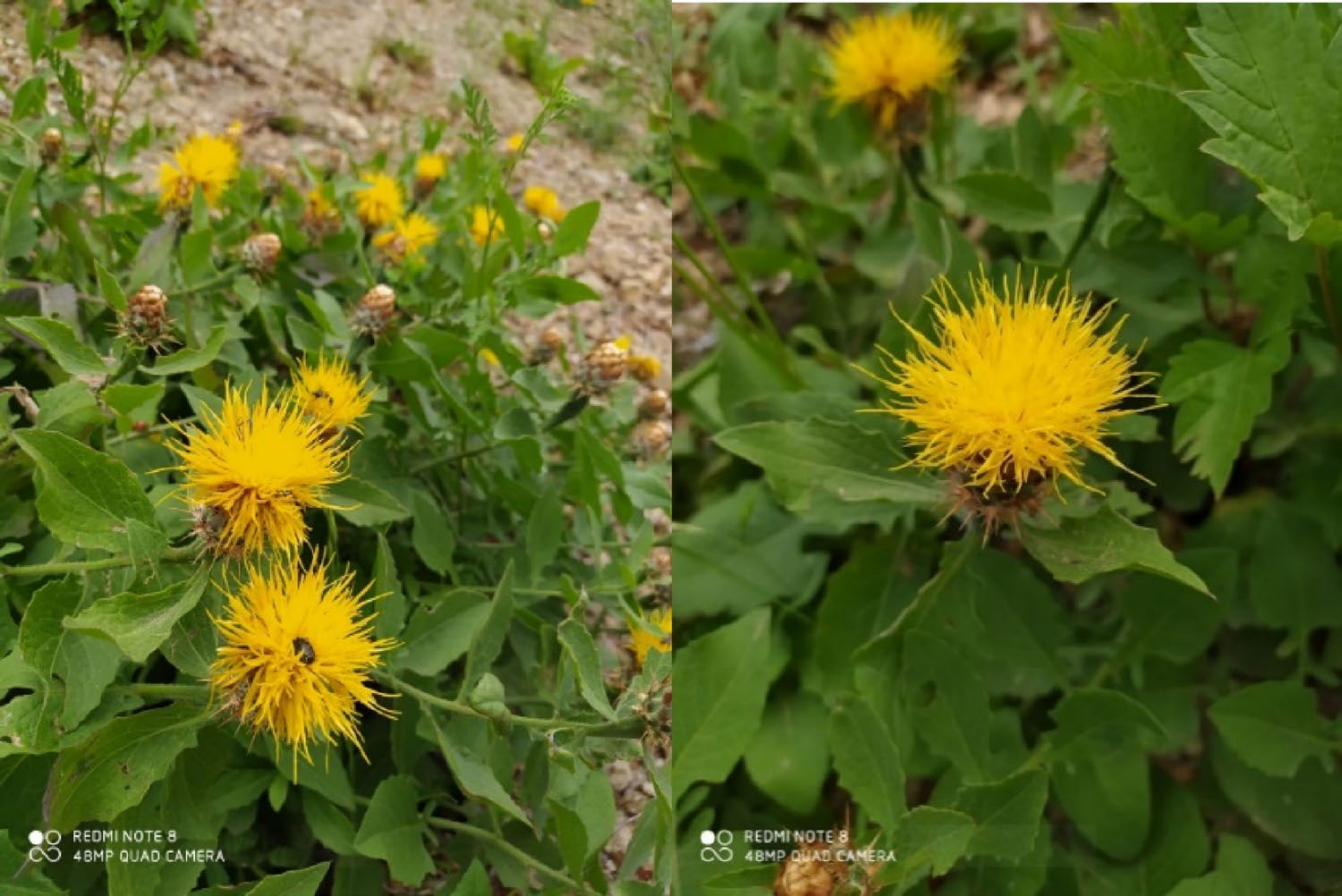
*Centaurea lycopifolia* plant

In the present work, the analgesic and anti-inflammatory effects of the *Centaurea lycopifolia* Boiss et Kotschy extract were elucidated. It is an endemic *Centaurea* species distributed in the Mediterranean Regions of Turkiye and the infusions and extracts prepared from the leaves of the plant are used for wounds and cuts (Ertas et al. 2014; Bülent Köse et al. 2016; Uysal et al. 2021). Plant’s primary active chemical constituents were identified using LC-MS/MS and analgesic and anti-inflammatory effects were investigated in mice and rats by developing an oral microemulsion formulation with the prepared extract. The antinociceptive effect of the prepared formulation was examined with Hot-Plate (HP) and Tail-Flick (TF) tests, and its anti-inflammatory effect was evaluated with Randall-Selitto and Plethysmometer tests. With this oral microemulsion formulation prepared with biocompatible materials, sustained drug release and low side effects, high solubility, and bioavailability will occur (Fig. 1). 

## Materials and methods

### Plant material

The above-ground parts of the *C. lycopifolia* (CL) plant were collected from Kahramanmaraş in June 2020 and May 2021. Kahramanmaraş has mountains and plains and is located in the southeastern part of Anatolia in Turkiye. It has a rich flora with approximately 2500 species and many native species (20%) because it is located in the Iran-Turanian and Mediterranean transition regions. Serpil Demirci Kayıran performed the species identification of the CL plant and recorded it in the Çukurova University Faculty of Pharmacy (Demirci and Özhatay 2012).

### Chemicals

Methanol was used for plant extraction. Carrageenan was used to induce inflammation in anti-inflammatory activity experiments. Aspirin was used for the control standard. Tween 80, Polyethylene Glycol 400 (PEG 400), and Isopropyl Myristate (IPM) were used for microemulsion formulation. They were acquired from Sigma Aldrich. All other chemicals and reagents used were of pharmaceutical and analytical grade.

### Preparation of the extracts

The aerial parts of the plant were dried at room temperature in the shade. The dried plant material was powdered using a laboratory-type electric blender and weighed. The powdered sample was mixed with methanol and subjected to maceration at room temperature for 24 h with the aid of a shaker, followed by extraction. The resulting dark green extract was filtered, and the methanol was removed under vacuum using a rotary evaporator. The obtained extract was stored at 4 °C for further use. Preliminary phytochemical screening was conducted to identify the phenolic compounds in the extracts (Ersoy et al. 2024).

### Determination of chemical content in plant extract

Phenolic acids and flavonoids in the prepared methanol extract were determined using a Shimadzu LCMS-8040 model tandem mass spectrometer. A quantitative evaluation of 53 compounds was performed using LabSolutions software (Shimadzu) according to method Yilmaz 2020. Chemical content analyses were made at Dicle University Science and Technology Application and Research Center.

### Oral microemulsion formulation evaluation

#### Preparation of microemulsion

For a good characterization of microemulsion systems, it is important to determine the pseudoternary phase diagram. This diagram describes the ideal experimental conditions in which the components have to be put together to form a clear preparation. The surfactant and cosurfactant were combined in different weight ratios (2:1, 3:1, and 4:1) and stirred with a magnetic stirrer to ensure a thorough mixing at 25 °C. The Oil and Smix (surfactants and cosurfactant) mixtures were titrated with double distilled water while being stirred until transparent. The microemulsions were stored in aluminum foil-wrapped dark-brown bottles for protection (Çevikelli et al. 2020; Güven et al. 2021).

The pseudoternary phase diagram was created using a computer program named Triangle Phase Diagram Analysis Software and all trials were replicated three times (Siafaka et al. 2021). The area generated by these points was understood to be the microemulsion area (Fig. 3). The largest percentage area of microemulsion in the majority of the phase diagrams occurred with the surfactant and cosurfactant weight ratio of 2:1. Formulations containing the extracts were prepared using the same technique (Kayiran et al. 2025a, b, c). The extract incorporation was assessed based on complete solubilization within the surfactant–oil system and the absence of precipitation or phase separation and the formulation was visually inspected and monitored for clarity, phase separation, or precipitation immediately after preparation and during the stability study period.

#### Physical stability of formulation

The selected formulations were subjected to the thermodynamic stability tests, including the centrifugation test and heating–cooling cycle. Thermodynamic stability was assessed by the procedure that three cycles between 4 °C and 40 °C with storage at each temperature for 48 h were studied. Also, the formulations were centrifuged at 3500 rpm for 30 min (Liu et al. 2012; Ozturk and Güven 2019; Maroof et al. 2023).

#### Characterization study of formulation

The pH values of blank and optimum microemulsion were quantified by measuring their pH values. These measurements were conducted in triplicate at room temperature using a pH meter electrode (WTW Profi Lab. pH 597, Germany). The results were reported as mean ± SD (Ozturk and Güven 2019).

The droplet size and polydispersity index of the microemulsion were examined at 25 °C by dynamic light scattering (Nano ZS, Malvern, UK). The mean droplet size (Z-average diameter) was calculated and recorded as mean diameter ± standard deviation (SD). Disposable plain folded capillary zeta cells from Malvern Nano ZS were used to determine the zeta potential, employing the system’s software. Each formulation was tested three times (Çevikelli et al. 2024; Güven et al. 2021).

#### Morphological analysis

A high-resolution inverted microscope (Leica DM IL LED Fluo, Germany) was used to examine the microscopic image to determine the microemulsions’ morphology (Kayiran et al. 2025a, b, c). The results were represented as corresponding figures.

### Pharmacological experiments

#### Animal

Male Swiss albino mice (30–40 g) and male Wistar rats (270–330 g) obtained from the Çukurova University Health Sciences Experimental Research Centre were used in the study. Mice were used in HP and TF tests and rats were used in Plethysmometer and Randall-Selitto tests. For evaluation of anti-inflammatory and analgesic effect, inflammation model was induced by carrageenan injection method (Hansra et al. 2000; Pereira et al.,^ 2024^). Experiments were carried out in behavioural laboratory standard conditions and in accordance with the Çukurova University Health Sciences Experimental Research Centre ethical guidelines. This study is approved by Çukurova University Animal Experiments Local Ethics Committee (TYL-2021-13381). Control excipient, aspirin as a standard anti-inflammatory analgesic, and CL extract formulations (400, 600, and 800 mg/kg dose) groups were set.

#### Evaluation of analgesic activity

##### Hot-plate test

The HP test (Hot/Cold Plate Model-D537) was used to evaluate the central analgesic effect. The modified test model defined by Eddy and Leimbach in 1953 was used (Sangha et al. 2011). The mouse was placed on the plate at 55 ± 1 °C. A nociceptive response such as a hind paw lick, a hind paw flick, or a jump was recorded as HP latency time (HPLT, sec). The animals that did not show any of these behaviours within 20 s (cut-off time) were taken from the test device and the latency time was recorded as 20 s. The test was performed before and 120 min after the administration of the excipient/drugs. The increase in latency time in the CL formulation groups was compared with the control and aspirin groups (Lende et al. 2011; Sun et al. 2018; Zhao et al. 2018).

##### Tail-flick test

The TF test described by D’Amour and Smith was used to evaluate spinal antinociceptive and analgesic activity (D’Amour and Smith 1941). The mouse was placed on the TF device (Harvard apparatus MPN 52-9495), and the light source was focused on the animal’s tail. The time from focusing the light on the tail to the animal pulling the tail away was determined and recorded as TF latency time (TFLT, sec). In order to preserve tail integrity, the animals that did not react within 10 s were taken off the device, and the reaction time was recorded as 10 s. The test was performed before and 120 min after the administration of the excipient/drugs. The increase in latency time in the CL formulation groups was compared with the control and aspirin groups (Saha et al. 2007; Mondal et al. 2019).

#### Evaluation of anti-inflammatory activity

##### Plethysmometer test

The method developed by Winter et al. is designed for the sensitive and rapid assessment of paw edema in rodents (Winter et al. 1962). The Digital Water Plethysmometer consists of two interconnected tubes filled with a conductive solution. By immersing the animal’s paw in the measuring tube, water displacement is produced in the second tube, altering the conductivity between the two platinum electrodes. The difference between inflamed and non-inflamed paw volumes indicates the lesion volume (ml) (Öztürk and Özbek 2005; Attia et al. 2016). Carrageenan was used to induce inflammation. Before the application of test materials, the right hind paw volume of the animal was measured. Then, excipient/drugs were administered to the control, aspirin, and extract groups via gavage 60 min before 0.1 ml of 1% carrageenan was injected into the animal’s right hind paw sub-plantar region. The paw volume of the mice was measured before carrageenan injection and after 15 and 120 min.

##### Randall-Selitto (analgesimeter) test

The Randall-Selitto, or paw pressure test, is often used to evaluate mechanical hyperalgesia. This test applies uniformly increasing pressure to the paw to determine the animal’s threshold response to pain (Deuis et al. 2017). In this study, this test was applied to measure the nociceptive effect due to inflammation using a Randall-Selitto analgesimeter (Ugo-Basile 37215, Comerio VA, Italy). The plantar surface of the rat’s hind paw was placed between the pointed probe (1 mm in diameter) and the flat surface of the device. The pressure was then increased by pressing a pedal at a constant rate until a nociceptive response was observed in the animal (Kayser 2013; Deuis et al. 2017). An injection of carrageenan was administered 30 min after the application of the excipient/drug to the control, aspirin, and extract formulation (400, 600, and 800 mg/kg) groups in the sub-plantar region of the paws. The Randall-Selitto test was performed before and 15 and 120 min after the carrageenan injection.

#### Groups and drug administration

Animals were divided into five groups (*n* = 10): the control excipient (drug/extract free microemulsion, control group), aspirin as a standard anti-inflammatory-analgesic agent (300 mg/kg, Aspirin group), and the CL formulations of 400, 600, and 800 mg/kg orally (CL groups). Aspirin and CL extract formulations were administered by gastric gavage. Carrageenan (0.1 ml of 1%) was injected into the right hind paw sub-plantar region of the animals.

### Statistical analysis

Measured values were calculated as mean and standard deviation. The assumption of a normal distribution was assessed using the Shapiro-Wilk test. Repeated measures analysis was used to compare the change in follow-up measures over time. The Kruskal-Wallis test was used for the overall comparison of the percentage changes at follow-up between the groups. The Mann-Whitney U test with Bonferroni correction was used for pairwise comparisons of the groups for cases found to be significant. Statistical analysis of the data was performed using the IBM SPSS Statistics Version 20.0 package program. The significance level was set as 0.05 for all tests.

## Results and discussion

*Centaurea lycopifolia* Boiss. et Kotschy. is an endemic Centaurea species belonging to the Centaurea genus and distributed in the Mediterranean regions of Turkiye. It exhibits various pharmacological activities due to the presence of numerous active compounds in its extract. Formulating the herbal material in a nanotechnological carrier like a microemulsion will make it suitable for pharmaceutical use. Additionally, its analgesic and anti-inflammatory effects were investigated for use in common symptoms of rheumatoid arthritis, by the carrageenan-induced inflammation model.

### Determination of chemical content in plant extract

**Fig. 2:**
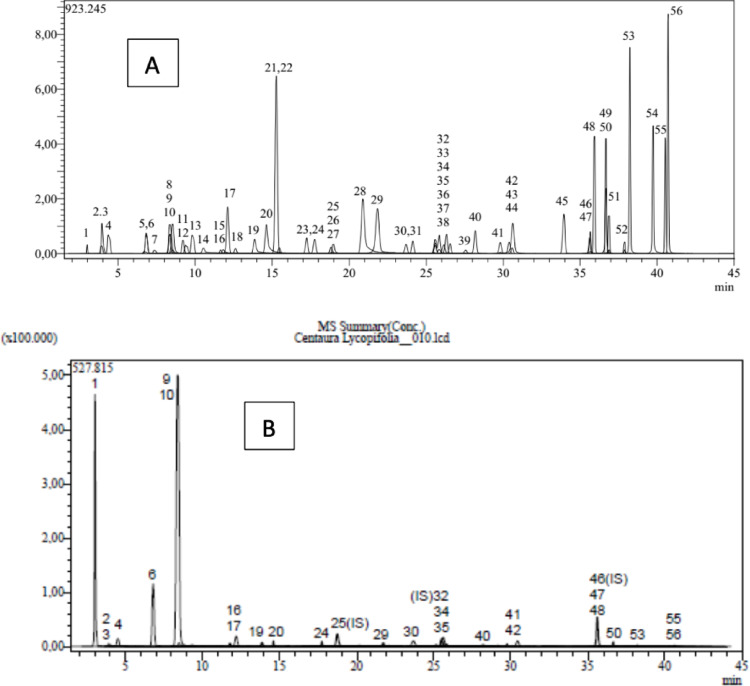
LC-ESI-MS/MS chromatograms of the standards **A** and the extract **B**.

LC-MS/MS chromatograms of the standards (A) and the extract (B) are given in Fig. 2. According to the results presented in Tables 1 and 25 different compounds were present in the extract, and the major compounds were quinic acid (31672 ± 1178.20 µg/g extract), chlorogenic acid (10867 ± 231.47 µg/g extract), and protocatechuic acid (3673 ± 145.45 µg/g extract). Furthermore, isoquercitrin (742 ± 16.32 µg/g extract), fumaric acid (493 ± 4.49 µg/g extract), astragalin (460 ± 5.24 µg/g extract), gallic acid (425 ± 4.76 µg/g extract), vanillic acid (187 ± 2.71 µg/g extract), cynaroside (135 ± 4.94 µg/g extract), caffeic acid (120 ± 1.82 µg/g extract), hesperidin (72 ± 2.41 µg/g extract), p-coumaric acid (52 ± 1.00 µg/g extract), salicylic acid (52 ± 0.82 µg/g extract), quercetin (45 ± 0.79 µg/g extract), quercitrin (39 ± 1.05 µg/g extract), vanillin (34 ± 0.41 µg/g extract), protocatechuic aldehyde (34 ± 1.35 µg/g extract), aconitic acid (33 ± 0.82 µg/g extract), luteolin (23 ± 0.72 µg/g extract), syringic aldehyde (15 ± 0.32 µg/g extract), acacetin (11 ± 0.40 µg/g extract), cosmosiin (10 ± 0.08 µg/g extract), apigenin (4 ± 0.07 µg/g extract), naringenin (3 ± 0.11 µg/g extract) and chrysin (2 ± 0.06 µg/g extract) were detected in the extract. There are only two studies in the literature regarding the chemical content of CL. One of these was the study of Zengin et al. and caffeoylquinic acid and vitexin compounds were found (Zengin et al. 2019). In another study, quinic acid, chlorogenic acid, malic acid, tr-aconitic acid, gallic acid, protocatechuic acid, tannic acid, tr-caffeic acid, vanillin, p-coumaric acid, rosmarinic acid, rutin, hespridin, hyperoside, 4-hydroxy benzoic acid, salicylic acid, coumarin, luteolin, apigenin, rhamnetin, and chyrisin compounds were detected, which is very similar to the present study (Boğa et al. 2016).

Quinic acid is a cyclohexanecarboxylic acid that is synthesized and derived from plants and is one of the major compounds determined in the studied extract. Quinic acid has many biological activities including antibacterial, antioxidant, antiviral, antidiabetic, anticancer, antinociceptive, analgesic, aging, and protective effects (Benali et al. 2024). Chlorogenic acid is a compound composed of coffee acid and quinic acid, is an effective component of many foods, is a phenolic compound found in many plants, fruits, and vegetables, and exhibits good analgesic and anti-inflammatory effects (Dos Santos et al. 2006; Liu et al. 2016; Singh et al. 2023). Protocatechuic acid (3,4-dihydroxybenzoicacid) is a phenolic compound that has recently garnered interest due to its biological effects. It can easily cross the blood-brain barrier. Thus, it is intriguing for its ability to inhibit neurodegenerative progression. Additionally, protocatechuic acid shows antihyperglycemic, anti-inflammatory, and analgesic effects in different animal models (Dikmen et al. 2019). The analgesic and anti-inflammatory effects of CL could be from the high amounts of quinic acid, chlorogenic acid, and protocatechuic acid found in the extract and their synergistic effects.

The chemical content analysis of the methanol extract of the CL plant by LC-MS/MS determined that the plant contained high levels of phenolic acids and flavonoids. Quinic acid, chlorogenic acid, protocatechuic acid, fumaric acid, aconitic acid, and gallic acids were high in phenolic acids. In previous studies on the plant, essential oil analyses were conducted and the presence of fatty acids such as caryophyllene oxide, spathulenol, widdrol, and -pinene were determined (Djeddi et al. 2011). The biological and pharmacological effects of phenolic acids have received attention recently. Chlorogenic acid is the most abundant isomer of the known caffeoylquinic acid isomers. In green coffee extracts and tea, it is one of the most abundant phenolic acid compounds. Chlorogenic acid is a biologically active polyphenol with antioxidant, antibacterial, hepatoprotective, cardioprotective, anti-inflammatory, antipyretic, and neuroprotective activities. It also has anti-obesity, antiviral, anti-microbial, anti-hypertension, free radical scavenger, and central nervous system stimulant activities. Additionally, chlorogenic acid modulates lipid metabolism and glucose in both genetic and healthy metabolic-related disorders (Naveed et al. 2018; Yan et al. 2022). The mechanism by which quinic acid and its esters exert their anti-inflammatory activities is unclear but appears to be related to the inhibition of the pro-inflammatory transcription factor nuclear factor kappa B (NF-kB) (Zamani-Garmsiri et al. 2022).

Phenolic acids such as quinic acid, chlorogenic acid, protocatechuic acid, vanillin, hesperidin, fumaric acid, vanillic acid, caffeic acid, syringic acid, and p-coumaric acid had the highest concentrations in CL plant extract using LC/MS-MS.

**Table 1:** Phenolic acids and flavonoid contents of CL extract (µg analyte/g extract)

Analytes	RT^a^	M.I. (m/z)^b^	F.I. (m/z)^c^
Quinic acid	3.0	190.8	93.0
Fumaric aid	3.9	115.2	40.9
Aconitic acid	4.0	172.8	129.0
Gallic acid	4.4	168.8	79.0
Epigallocatechin	6.7	304.8	219.0
Protocatechuic acid	6.8	152.8	108.0
Catechin	7.4	288.8	203.1
Gentisic acid	8.3	152.8	109.0
Chlorogenic acid	8.4	353.0	85.0
Protocatechuic aldehyde	8.5	137.2	92.0
Tannic acid	9.2	182.8	78.0
Epigallocatechin gallate	9.4	457.0	305.1
1,5-dicaffeoylquinic acid	9.8	515.0	191.0
4-OH Benzoic acid	10.5	137,2	65.0
Epicatechin	11.6	289.0	203.0
Vanilic acid	11.8	166.8	108.0
Caffeic acid	12.1	179.0	134.0
Syringic acid	12.6	196.8	166.9
Vanillin	13.9	153.1	125.0
Syringic aldehyde	14.6	181.0	151.1
Daidzin	15.2	417.1	199.0
Epicatechin gallate	15.5	441.0	289.0
Piceid	17.2	391.0	135/106.9
*p*-Coumaric acid	17.8	163.0	93.0
Ferulic acid-D3-IS^*h*^	18.8	196.2	152.1
Ferulic acid	18.8	192.8	149.0
Sinapic acid	18.9	222.8	193.0
Coumarin	20.9	146.9	103.1
Salicylic acid	21.8	137.2	65.0
Cynaroside	23.7	447.0	284.0
Miquelianin	24.1	477.0	150.9
Rutin-D3-IS^*h*^	25.5	612.2	304.1
Rutin	25.6	608.9	301.0
isoquercitrin	25.6	463.0	271.0
Hesperidin	25.8	611.2	449.0
*o*-Coumaric acid	26.1	162.8	93.0
Genistin	26.3	431.0	239.0
Rosmarinic acid	26.6	359.0	197.0
Ellagic acid	27.6	301.0	284.0
Cosmosiin	28.2	431.0	269.0
Quercitrin	29.8	447.0	301.0
Astragalin	30.4	447.0	255.0
Nicotiflorin	30.6	592.9	255.0/284.0
Fisetin	30.6	285.0	163.0
Daidzein	34.0	253.0	223.0
Quercetin-D3-IS^*h*^	35.6	304.0	275.9
Quercetin	35.7	301.0	272.9
Naringenin	35.9	270.9	119.0
Hesperetin	36.7	301.0	136.0/286.0
Luteolin	36.7	284.8	151.0/175.0
Genistein	36.9	269.0	135.0
Kaempferol	37.9	285.0	239.0
Apigenin	38.2	268.8	151.0/149.0
Amentoflavone	39.7	537.0	417.0
Chrysin	40.5	252.8	145.0/119.0
Acacetin	40.7	283.0	239.0

^*a*^R.T.: Retention time, ^*b*^MI (*m/z)*: Molecular ions of the standard analytes (m/z ratio), ^*c*^FI (*m/z)*: Fragment ions.

### Preparation and evaluation of oral microemulsion formulation

**Fig. 3:**
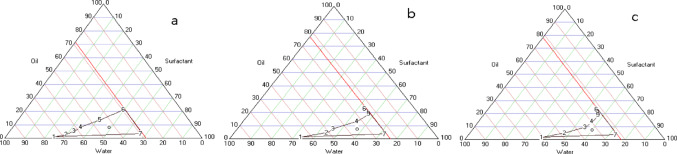
Pseudoternary phase diagrams of microemulsion formulations (**a**:2:1,** b**:3:1,** c**:4:1)

By using different surfactants and cosurfactants in a preformulation study, the ideal microemulsion system was determined. Tween 80 was chosen as a surfactant because of its biodegradable, biocompatible, and non-ionic nature. It is also a GRAS (Generally Recognized as Safe) excipient commonly used in pharmaceutical formulations (Yusakul et al. 2024). The microemulsion study with Melaleuca alternifolia Chell extract indicated that using isopropyl myristate as the oil phase facilitated the formation of transparent and fluid systems when high amounts of Tween 80 were used. Additionally, the study found that microemulsion regions with PEG 400 as a cosolvent required less surfactant compared to those with propylene glycol (Oliveira et al. 2021). Tween 80, a non-ionic surfactant with a favorable oral safety profile, is frequently used to stabilize oil-in-water microemulsions and enhance drug solubilization. PEG 400 is commonly incorporated as a co-surfactant or co-solvent to reduce interfacial tension, expand the microemulsion region, and maintain formulation homogeneity. Similar combinations of IPM, Tween-type surfactants, and PEG 400 have been successfully applied in oral delivery systems for herbal extracts and hydrophobic compounds, supporting their suitability for the present formulation (Liı et al., 20212). After the preformulation studies, the areas for triangular phase diagram drawing were narrowed, and microemulsion areas were drawn over six formulation points (Ozturk and Güven 2019; Güven et al. 2021). To draw a triangular phase diagram, the amounts of oil, surfactant, and water were calculated as percentages and presented. For the ideal microemulsion formulation, IPM in the oil phase, Tween 80 as the surfactant, and PEG 400 as the cosurfactant were selected. Surfactant and cosurfactant ratios were separately weighed as 2:1, 3:1, and 4:1 (Fig. 3).

Among the various ratios tested, Tween 80: PEG 400 (2:1) produced the largest microemulsion region areas. This formulation was used in the next experiments (Table 2). In microemulsion-based systems, the concept of extract incorporation differs fundamentally from particulate carriers, as these formulations represent thermodynamically stable, isotropic single-phase systems in which the active components are molecularly solubilized within the surfactant–oil matrix. Consequently, successful incorporation is evaluated through physicochemical indicators such as complete solubilization, formulation clarity, and the absence of precipitation or phase separation. When examined under a strong light, the microemulsion containing blank or extract was clear, transparent, liquid, single phase, had no drug precipitation, and a homogeneous appearance. In the study by Panapisal et al. (2012), silymarin was successfully incorporated into a microemulsion system. The formulations were visually inspected immediately after preparation and during storage, and they remained clear without any signs of precipitation or phase separation. This indicates that silymarin was fully solubilized within the system and that the formulation maintained its physical stability, directly supporting the argument that extract incorporation should ensure clarity and absence of instability phenomena.

**Table 2:** Optimum formulation ingredients and the physical properties of the formulations (*n* = 3)

Formulation (% w/w)	IPM	Tween 80	PEG 400	Distilled water	Extract	pH ±SD	Droplet size ± SD (nm)	PDI ±SD	Zeta potential ± SD (mV)
Blank	8.72	32.22	16.11	42.95	–	5.42 ± 0.1	286.300 ± 4.920	0.415 ± 0.3	−0.815 ± 0.03
Active substance	8.72	32.22	16.11	32.95	10	5.66 ± 0.1	262.400 ± 4.660	0.495 ± 0.3	−0.520 ± 0.05

The aqueous solubility of herbal extracts is generally low and they are poorly absorbed, and lack stability. Microemulsion is a thermodynamically stable, transparent and convenient system with the ability to improve the solubility and stability of these phytochemicals. Particularly for dermal or transdermal use, microemulsions facilitate skin penetration and bioavailability because of their droplet size and high surface area. Moreover, microemulsions are advantageous in herbal extract delivery due to their ability to contain both hydrophilic and lipophilic components, and their compositional variability (Calvo-Castro et al. 2023; Leanpolchareanchai and Teeranachaideekul 2023).

**Fig. 4:**
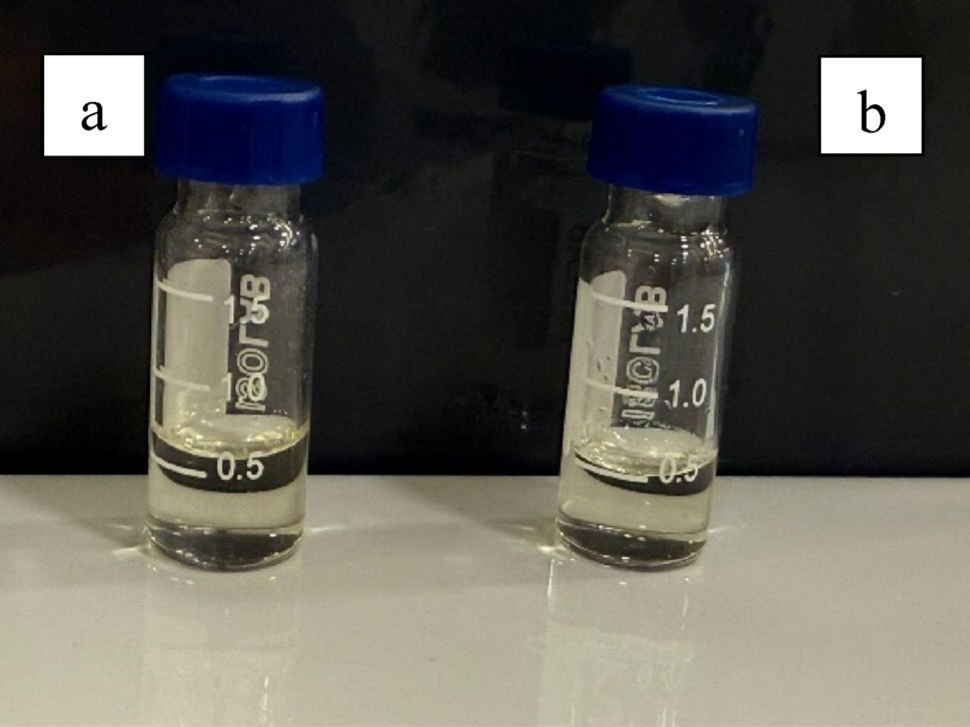
Physical appearance of the ideal microemulsion formulation before **a** and after **b** centrifugation test

In almost all microemulsion studies, the physical stability is checked first. The formulation prepared for this purpose is subjected to centrifugation at a certain time and speed and visually inspected for phase separation. In this study, as shown in Fig. 4, no visible phase separation, precipitation, or sedimentation was observed after centrifugation, confirming the physical stability of the microemulsion. These findings are consistent with commonly employed stability assessment approaches reported for microemulsion systems. In another study, the physical stability of the microemulsion was examined by centrifuging at different speeds for 30 min at regular intervals during storage and the phase separation was examined. In addition, the colour change and clarity were visually recorded. Finally, the formulation was stressed for two days using the heating and cooling method, and its physical state was examined after it was brought to room temperature. A lack of change during storage in all these processes showed that the formulation was physically stable (Patel and Sawant 2009). In another study, a microemulsion formulation was developed, and its physical stability was assessed using centrifugation and optical brightness study. It was stated that upon the addition of the drug to the novel microemulsion, there was no alteration in the microstructure, and all formulations remained stable and transparent for approximately 8 months (Mujahid et al. 2024).

Stress testing is crucial to eliminate the risk of forming nonstable formulations. In the study conducted by Maroof et al., thermodynamic stability testing was performed using heating-cooling cycles, centrifugation tests, and freeze-thaw cycles (Maroof et al. 2023). The propolis loaded microemulsion formulation demonstrated strong physical stability during thermodynamic testing, showing no signs of phase separation, turbidity, creaming and returning to its original state after stress removal. The absence of phase separation, turbidity, creaming in the test results indicates a positive outcome for the formulation’s shelf life. When the formulations containing both placebo and extraction prepared for use in in vivo studies were examined in terms of physical stability, they did not undergo any change.

The microemulsions were confirmed through characterization studies. The results of the measurements of pH values, the droplet sizes, polydispersity indexes (PDI) and zeta potentials of microemulsions are represented in Table 2. The excipients used determine the pH value also can affect the zeta potential. The gastrointestinal system can tolerate a wide range of pH values (pH 2.0–7.0). The observed pH values were found to be suitable for oral administration (Maroof et al. 2023; Köksal Karayıldırım et al. 2024).

Droplet size is an important factor in nanocarriers like microemulsion, as it significantly impacts the rate and extent of drug release or absorption. A laser particle size analyser was used to measure droplet size and distribution (Wang et al. 2022). The small droplet size and narrow PDI are not only desirable for ensuring stability and reproducibility but also contribute to improving the solubility and subsequent bioavailability of the active agent. In addition to stability, small droplet size also facilitates uptake by the gastrointestinal system (Shevalkar and Borse 2024). The distribution of the drug in nanometer-sized droplets as a solution improves the dissolution rate in the surrounding aqueous phase and typically leads to enhanced drug bioavailability in vivo. The prepared microemulsion had a relatively larger droplet size than required to be characterized as a microemulsion. However, the obtained values are considered sufficient in terms of the formulation’s stability and applicability.

The droplet size of the blank formulation was measured as 286.3 ± 4.9 nm, whereas the extract-loaded formulation exhibited a smaller mean droplet size of 262.4 ± 4.7 nm. This indicates a measurable reduction in droplet size upon incorporation of the plant extract. The slight reduction in droplet size after incorporation of the plant extract may be attributed to its amphiphilic phenolic constituents, which can lower interfacial tension and promote tighter interfacial packing. Phenolic residues are known to position themselves at the oil–water interface due to their hydrophilic and hydrophobic moieties, thereby enhancing emulsion stability. Lignin-derived phenolics, in particular, can associate with droplet surfaces and contribute to the formation of smaller and more stable droplets. Consistent with this mechanism, another study reported that phenolic-rich extracts obtained from avocado waste reduced interfacial tension and produced emulsions with significantly smaller droplet sizes compared with blank formulations (Lehtonen et al. 2018; Iymen et al. 2020).

PDI is a parameter used to describe droplet size. PDI is a dimensionless parameter used to assess droplet size uniformity; values below 0.5 indicate uniformity, whereas values exceeding 0.7 reflect a broad size distribution, rendering the sample unsuitable for dynamic light scattering analysis (Ruiz et al. 2022). A study highlighted that a high polydispersity index could affect the stability of microemulsions, but emulsions should reform upon resuspension, and separation issues could be resolved by increasing viscosity. In our study, the PDI values were also relatively high; however, they are not at a level that would affect stability (Alkrad et al. 2022). There was no significant change in the particle size and PDI for both the blank and extract loaded formulations, indicating good stability.

According to the results obtained, the zeta potential of the formulation was found to be close to zero. A previous study concluded that the use of non-ionic surfactants reduces the zeta potential value (Koli et al. 2021). The zeta potential provided consistent results in relation to the surfactant and co-surfactant used.

**Fig. 5:**
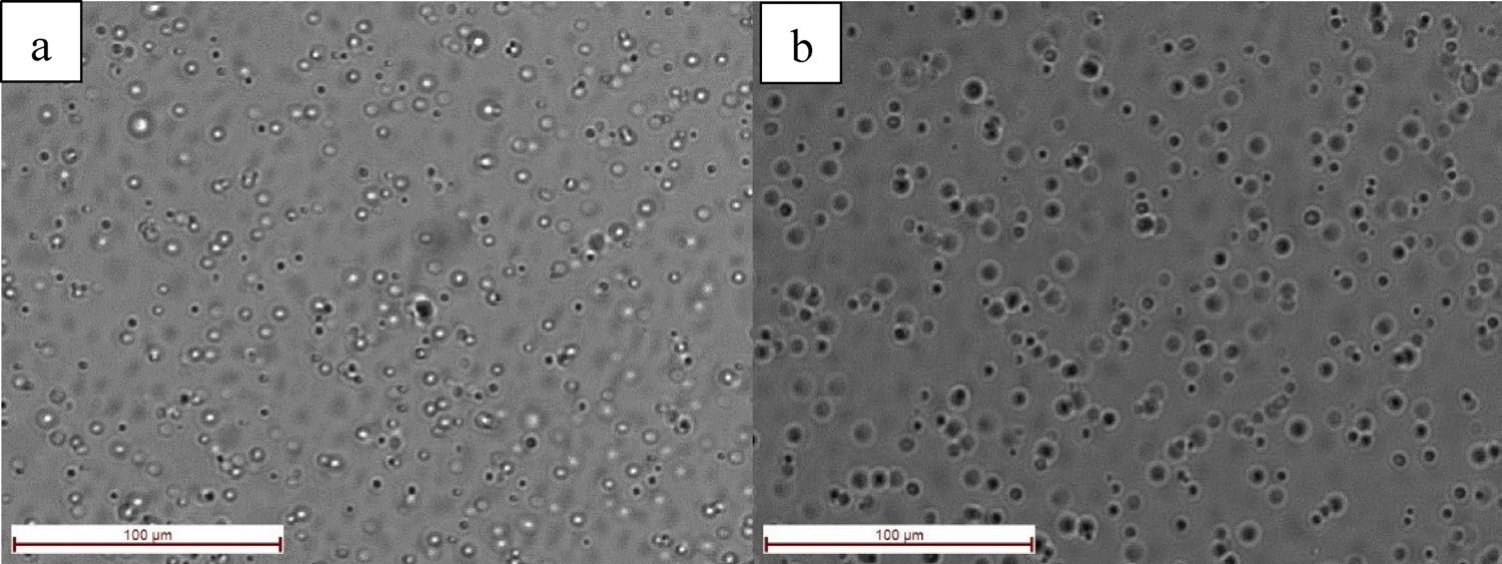
Morphological analysis of blank **a** and extract containing **b** microemulsion formulations using high-resolution microscopy

A direct imaging method called high-resolution microscopy was used to identify the surface morphology of the microemulsion formulations, to evaluate fundamental properties including size, shape, and aggregation state. The blank and the extract containing emulsions’ microscopic images showed that globular drops with distinct edges were present (Fig. 5) (Kayiran et al. 2025a, b, c).

### Pharmacological experiments

#### Evaluation of analgesic activity

Rheumatic diseases negatively affect patients’ quality of life and daily work efficiency. Inflammation in the tissues causes tissue damage and pain in rheumatic diseases. For this reason, anti-inflammatory and analgesic drugs have an important place in rheumatic disease treatment. However, since the drugs used for rheumatic diseases cannot provide complete treatment, new therapies are being developed. Herbal medicines are a source of raw materials in both traditional and modern medicine. Several species within the *Centaurea* genus have been reported to exhibit significant analgesic and anti-inflammatory activities, often attributed to their rich phenolic and flavonoid content. For instance, extracts of *Centaurea solstitialis*, *Centaurea calcitrapa*, and *Centaurea cyanus* have demonstrated notable inhibition in carrageenan-induced paw edema and nociceptive pain models, with effects partially comparable to standard non-steroidal anti-inflammatory drugs.

In this study, we investigated whether the CL plant has analgesic and anti-inflammatory effects, based on its popular use as an antirheumatic. One of the most important symptoms of rheumatic diseases is pain (Atzeni et al. 2015). We used two pain models frequently used in the literature to investigate the antinociceptive effect of the formulation prepared from the plant extract. Pain models developed using experimental animals are based on the principle of measuring or observing the animal’s response after the application of a painful stimulus. In the models we used in the study, pain (nociception) was created by thermal stimulation in the animal, and the response time (latency time) of the animal to this stimulus was recorded in seconds. It is expected that the response time of the animal will be prolonged after the administration of the compounds whose antinociceptive effects were investigated. The HP test is preferred for the evaluation of central pain (Modi et al. 2023) and the TF test is preferred for the evaluation of spinal pain (Flecknell 2018). Thermal and radiant heat are used in the HP and TF tests, respectively (Santenna et al. 2019).

**Fig. 6:**
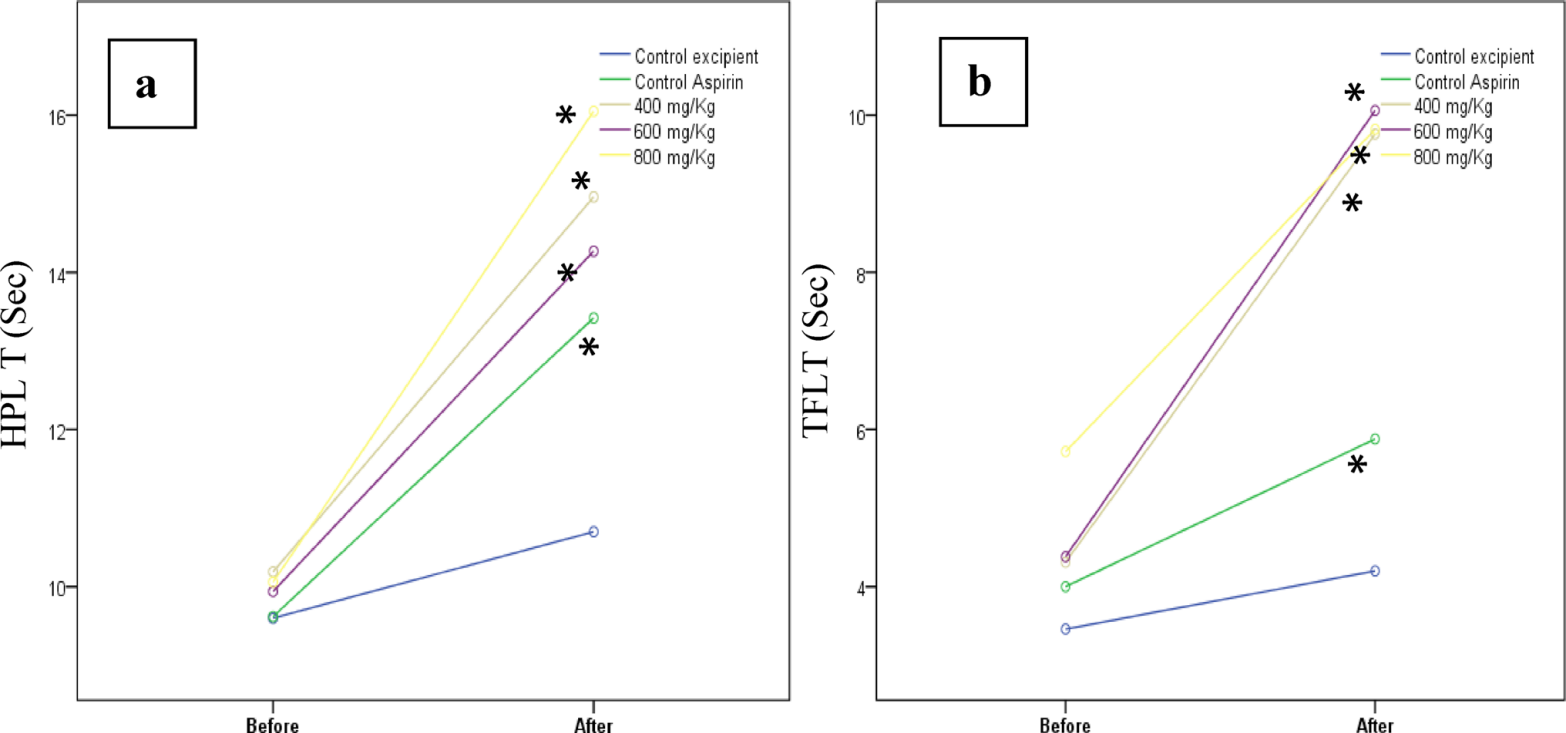
**a** HP latency times (HPLT) of the Control, Aspirin and CL extract formulation groups. **b** TF latency times (TFLT) of the Control, Aspirin and CL extract formulation groups (Before and after administration of drug/excipient). * Different from Control group (*p* < 0,05)

According to HP test results in this study, the CL plant extract reduced the experimental pain (nociception) induced by thermal stimuli. The antinociceptive effect of the extract was dose-dependent, as it showed similar effects at doses of 400 and 600 mg/kg, while the effect increased at 800 mg/kg. The effect at all three doses was greater than that of the standard drug aspirin, and the greatest difference was seen at 800 mg/kg (Fig. 6a). The HP test is a supraspinally organized response used in neuroscience to measure the reaction times of rats or mice when exposed to a heated metallic plate. It involves observing behaviours such as paw licking and jumping, which are supraspinally integrated responses (Modi et al. 2023). Our results show that CL extract may have a central analgesic action.

The TF test measures a reflexive, spinally mediated response to noxious stimulation (Bannon and Malmberg 2007). According to our TF test results, an antinociceptive effect was found in CL extract formulations. Unlike the HP test, the TF test antinociceptive effect was not dose dependent. On the other hand, the effect at all three doses was greater than that of the standard drug aspirin, and the greatest difference was seen with 600 mg/kg (Fig. 6b). The CL plant extract formulations are more effective than aspirin, which we used as a standard analgesic in these tests, suggesting that this plant may be a good analgesic drug candidate for development.

#### Evaluation of anti-inflammatory activity

**Fig. 7:**
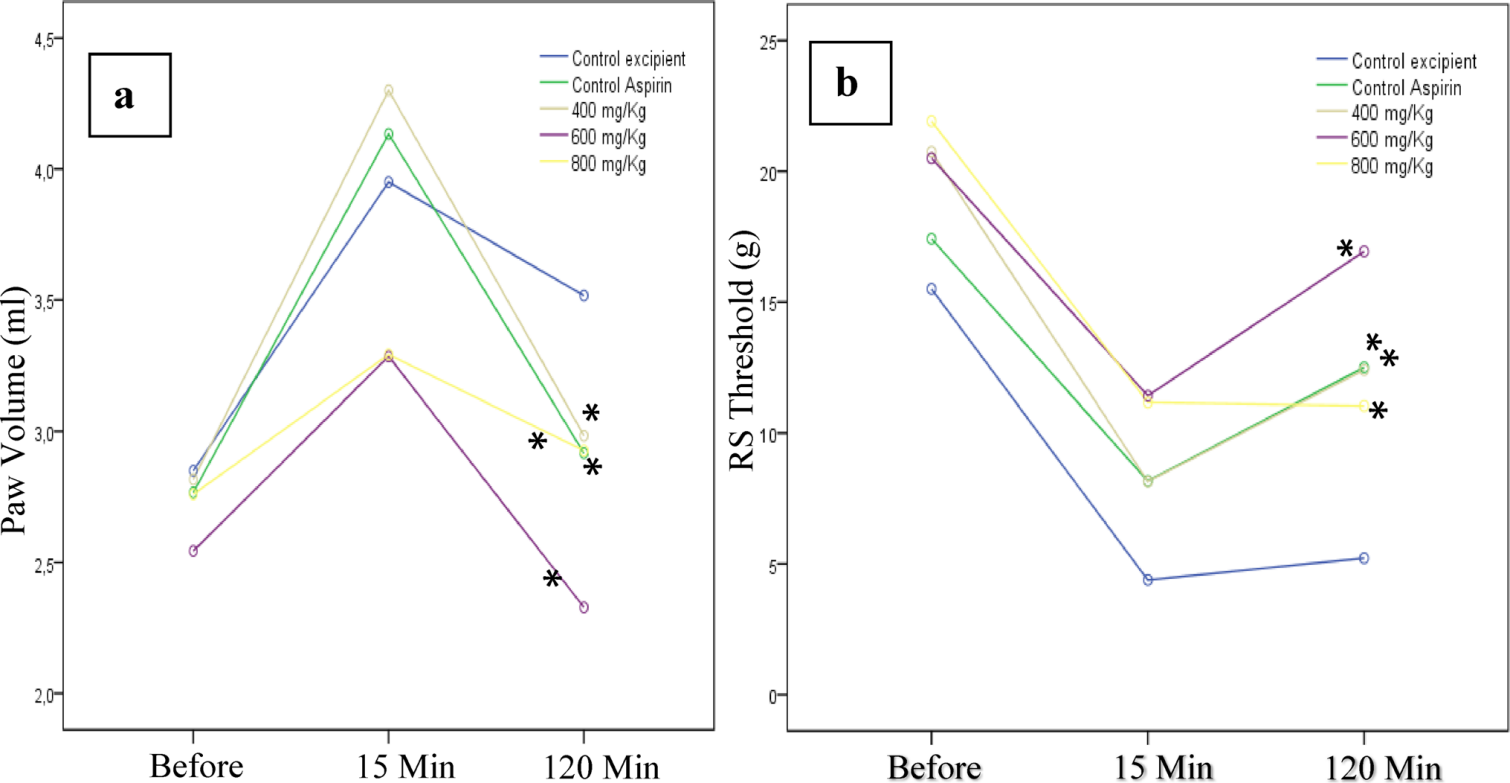
**a**. Paw volume (ml) of the Control, Aspirin and CL extract formulation groups** b**. Randall-Selitto Threshold (RS Threshold, weight in grams) of the Control, Aspirin and CL extract formulation groups (before and 15 and 120 min after the carrageenan injection). *Different from Control group (*p*<0,05)

In our study, we used the Plethysmometer test (Paw Edema) to evaluate the anti-inflammatory effect of the plant extract. The Plethysmometer test is widely used to measure the effectiveness of anti-inflammatory agents to reduce edema. This test measures the volume increase due to inflammation (Fereidoni et al. 2000). We used carrageenan to induce experimental edema in rat paws, which often is used in the testing of new NSAIDs. Edema was observed in all groups 15 min after the injection of carrageenan. While the volume increase in the control group did not show a significant change 2 h after the carrageenan injection, edema decreased significantly in the aspirin and CL groups. While similar efficacy to aspirin was observed at doses of 400 and 800 mg/kg CL, more efficacy than aspirin was found at 600 mg/kg CL (Fig. 7a).

We used the Randall-Selitto test to evaluate pain due to inflammation. The Randall-Selitto test is a standard test that measures the impact of analgesic agents on the tolerance to mechanical pressure stimulation to evaluate painful inflammatory responses (Anseloni et al. 2003). In inflamed tissue, there is increased sensitivity to pain (hyperalgesia). This test evaluates the hyperalgesia and the antihyperalgesic effect of the drug. The non-inflamed paw can endure more pressure, while the inflamed paw reacts earlier. Antihyperalgesic drugs prevent the inflamed paw from reacting or allow it to endure more pressure. Carrageenan was used to induce experimental edema. Fifteen minutes after the carrageenan administration, animals in all groups responded to a lower pressure than before the carrageenan administration. Two hours after the administration of carrageenan, the pressure to which the animals reacted increased in the aspirin group and the CL 400 mg/kg and 600 mg/kg groups. However, no effect was observed in the 800 mg/kg CL group. Compared to the control group, the 600 mg/kg formulation had the greatest effect on values measured after 2 h (Fig. 7b). This suggests that the CL extract formulation exhibits a maximum effect and does not show further effects as the dose is increased.

It has been indicated in previous studies that the anti-nociceptive action of NSAIDs might involve central neural mechanisms in addition to the peripheral analgesia induced by COX inhibition. It is also known that there is significantly greater supraspinal modulation of pain perception in the hot plate method compared to the tail flick method (Santenna et al. 2019). In this study, the CL extract had analgesic and anti-inflammatory effects comparable to aspirin. Thus, the CL plant might be a good candidate for analgesic and anti-inflammatory drug development studies. The analgesic and anti-inflammatory effects of CL are due to its high amounts of quinic acid, chlorogenic acid, and protocatechuic acid, which have anti-inflammatory and analgesic effects, and their synergistic effects (Wu et al. 2024). In future studies, the effectiveness of the active substances detected in the plant extract in the same tests can be examined, and it can be determined from which substance the effect arises. The effect of the extract may be due to one substance, or it may be the combined effect of several substances. This also needs to be investigated in future studies. And then, no formal acute or sub-acute toxicology study was conducted; however, no abnormalities in behavior, weight changes or visible side effects were observed in any of the animals throughout the experimental period. The oral microemulsion was generally well tolerated and did not result in distress or lethality in any of the animals. However, thorough in vivo toxicity testing are necessary to fully elucidate the safety of a *C. lycopifolia* extract loaded formulation and ought to be further explored in future studies. The pharmacological effects observed in the present study may be mechanistically linked to the modulation of key inflammatory and oxidative stress–related signaling pathways by phenolic acids present in the extract. Several phenolic acids, such as caffeic and ferulic acid, have been reported to inhibit cyclooxygenase (COX) activity, thereby reducing prostaglandin synthesis and inflammatory responses. Moreover, these compounds are known to suppress NF-κB activation, leading to the downregulation of pro-inflammatory cytokines including TNF-α and IL-6. In addition, the strong antioxidant capacity of phenolic acids may indirectly attenuate inflammation by reducing reactive oxygen species–mediated activation of inflammatory cascades. Although direct molecular assays were not performed in the current study, the observed pharmacological outcomes are consistent with these well-established mechanisms reported in the literature.

## Conclusion

Herbal medicines are a resource for raw materials in both traditional and modern medicine. The CL plant has been used to treat various ailments in people. This study was carried out to investigate the use of the plant as an antirheumatic and to show whether it has such an effect with preclinical studies using appropriate in vivo experimental models. One of the most important symptoms of rheumatic diseases is pain. For this reason, we used two pain models frequently used in the literature to investigate the antinociceptive effect of the formulation prepared from the plant extract.

When the both blank and extract containing formulations prepared for use in in vivo studies were examined in terms of physical stability, they did not undergo any change. Including both oil and water phases in the microemulsion formulation improves the solubility profile of the extract. Increasing the solubility property of the system might contribute positively to the bioavailability of the extract. A flow characteristic suitable for oral use and homogeneous behaviour such as existing in a single phase throughout the stability study shows that it will increase patient compliance during long-term use.

Pain models developed using experimental animals are based on the principle of measuring or observing the animal’s response after the application of a painful stimulus. In the models we used in the study, pain (nociception) is created by thermal stimulation in the animal, and the response time of the animal to this stimulus is recorded in seconds. It is expected that the response time of the animal will be prolonged after the administration of the compounds whose antinociceptive effects we have investigated. The models we used in our study were chosen because the HP test is preferred for the evaluation of central pain and the TF test is preferred for the evaluation of spinal pain. The change in reaction time (delay in reaction time) in the HP test at the 800 mg/kg extract formulation was 54.17%, while this value was 39.50% in aspirin and 11.45% in the control group. In the TF test, the extract showed an antinociceptive effect within a certain dose range. The effect at all three doses is greater than that of the standard drug aspirin, and the greatest difference was seen at the 600 mg/kg dose. In the 600 mg/kg extract formulation, the change in reaction time was 129.68%, while in aspirin this value was 47.00% and 21.38% in the control group.

All the findings show the CL plant is a good candidate for analgesic and anti-inflammatory drug development studies. The analgesic and anti-inflammatory properties of CL stem from the high amounts of quinic acid, chlorogenic acid, and protocatechuic acid, which have anti-inflammatory and analgesic effects, and their synergistic effects. In future studies, the effectiveness of the active substances detected in the plant extract in the same tests can be examined, and it can be determined from which substance the effect arises. The effect of the extract may be due to one substance, or it may be the combined effect of several substances. This also needs to be investigated in future studies. In addition, in this study, the composition of the microemulsion formulation in which the extract was prepared was also examined.

## Data Availability

The material and data of this study are available from the corresponding author on reasonable request.
